# A Maximum-Entropy Method to Estimate Discrete Distributions from Samples Ensuring Nonzero Probabilities

**DOI:** 10.3390/e20080601

**Published:** 2018-08-13

**Authors:** Paul Darscheid, Anneli Guthke, Uwe Ehret

**Affiliations:** 1 Institute of Water Resources and River Basin Management, Karlsruhe Institute of Technology—KIT, 76131 Karlsruhe, Germany; 2 Institute for Modelling Hydraulic and Environmental Systems (IWS), University of Stuttgart, 70569 Stuttgart, Germany

**Keywords:** histogram, sample, discrete distribution, empty bin, zero probability, Clopper–Pearson, maximum entropy approach

## Abstract

When constructing discrete (binned) distributions from samples of a data set, applications exist where it is desirable to assure that all bins of the sample distribution have nonzero probability. For example, if the sample distribution is part of a predictive model for which we require returning a response for the entire codomain, or if we use Kullback–Leibler divergence to measure the (dis-)agreement of the sample distribution and the original distribution of the variable, which, in the described case, is inconveniently infinite. Several sample-based distribution estimators exist which assure nonzero bin probability, such as adding one counter to each zero-probability bin of the sample histogram, adding a small probability to the sample pdf, smoothing methods such as Kernel-density smoothing, or Bayesian approaches based on the Dirichlet and Multinomial distribution. Here, we suggest and test an approach based on the Clopper–Pearson method, which makes use of the binominal distribution. Based on the sample distribution, confidence intervals for bin-occupation probability are calculated. The mean of each confidence interval is a strictly positive estimator of the true bin-occupation probability and is convergent with increasing sample size. For small samples, it converges towards a uniform distribution, i.e., the method effectively applies a maximum entropy approach. We apply this nonzero method and four alternative sample-based distribution estimators to a range of typical distributions (uniform, Dirac, normal, multimodal, and irregular) and measure the effect with Kullback–Leibler divergence. While the performance of each method strongly depends on the distribution type it is applied to, on average, and especially for small sample sizes, the nonzero, the simple “add one counter”, and the Bayesian Dirichlet-multinomial model show very similar behavior and perform best. We conclude that, when estimating distributions without an a priori idea of their shape, applying one of these methods is favorable.

## 1. Introduction

Suppose a scientist, having gathered extensive data at one site, wants to know whether the same effort is required at each new site, or whether already a smaller data set would have provided essentially the same information. Or imagine an operational weather forecaster working with ensembles of forecasts. Working with ensemble forecasts usually involves handling considerable amounts of data, and the forecaster might be interested to know whether working with a subset of the ensemble is sufficient to capture the essential characteristics of the ensemble. If what the scientist and the forecaster are interested in is expressed by a discrete distribution derived from the data (e.g., the distribution of vegetation classes at a site, or the distribution of forecasted rainfall), then the representativeness of a subset of the data can be evaluated by measuring the (dis-)agreement of a distribution based on a randomly drawn sample (“sample distribution”) and the distribution based on the full data set (“full distribution”). One popular measure for this purpose is the Kullback–Leibler divergence [1]. Depending on the particular interest of the user, potential advantages of this measure are that it is nonparametric, which avoids parameter choices influencing the result, and that it measures general agreement of the distributions instead of focusing on particular aspects, e.g., particular moments.

For the use cases described above, if the sample distribution is derived from the sample data via the bin-counting (BC) method, which is the most common and probably most intuitive approach, a situation can occur where a particular bin in the sample distribution has zero probability but the corresponding bin in the full distribution has not. From the way the sample distribution was constructed, we know that this disagreement is not due to a fundamental disagreement of the two distributions, but rather that this is a combined effect of sampling variability and limited sample size. However, if we measure the (dis-)agreement of the two distributions via Kullback–Leibler divergence, with the full distribution as the reference, divergence for that bin is infinite, and consequently so is total divergence. This is impractical, as an otherwise possibly good agreement can be overshadowed by a single zero probability. A similar situation occurs if a distribution constructed from a limited data set (e.g., three months of air-temperature measurements) contains zero-probability bins, but from physical considerations we know that values falling into these zero-probability bins can and will occur if we extend the data set by taking more measurements.

Assuring nonzero (NZ) probabilities when estimating distributions is a requirement found in many fields of engineering and sciences [2,3,4]. If we stick to BC, this can be achieved either by adjusting the binning to avoid zero probabilities [5,6,7,8,9], or by replacing zero probabilities with suitable alternatives. Often-used approaches to do so are (i) assigning a single count to each empty bin of the sample histogram, (ii) assigning a (typically small) preselected probability mass to each zero probability bin in the sample pdf and renormalizing the pdf afterwards, (iii) spreading probability mass within the pdf by smoothing operations such as Kernel-density smoothing (KDS) [10] (an extensive overview on this topic can be found in Reference [11]), and (iv) assigning a NZ guaranteeing prior in a Bayesian (BAY) framework. Whatever method we apply, desirable properties we may ask for are introducing as little unjustified side information as possible (e.g., assumptions on the shape of the full distribution) and, like the BC estimator, convergence towards the full distribution for large samples.

In this context, the aim of this paper is to present a new method of calculating the sample distribution estimate, which meets the mentioned requirements, and to compare it to existing methods. It is related to and draws from approaches to estimate confidence intervals of discrete distributions based on limited samples [12,13,14,15,16,17]. In the remainder of the text, we first introduce the “NZ” method and discuss its properties. Then we apply the NZ method and four alternatives to a range of typical distributions, from which we draw samples of different sizes. We use Kullback–Leibler divergence to measure the agreement of the full and the sample distributions. We discuss the characteristics of each method and their relative performance with a focus on small sample sizes and draw conclusions on the applicability of each method.

## 2. The NZ Method

### 2.1. Method Description

For a variable with discrete distribution p with K bins, and a limited data sample S, thereof of size n, we derive a NZ estimator $\hat{p}$ for p based on S as follows: For the occurrence probability of each bin ${ß}_{k}$ (k=1,…,K), we calculate a BC estimator $q_{k}$ and its confidence interval $CI_{p,k}=\left[p_{k,lower};p_{k,upper}\right]$ on a chosen confidence level (e.g., 95%).

Based on the fact that the occurrence probability of a given bin from n-repeated trials follows a binomial distribution with parameters n and $p_{k}$, there exist several ways to determine a confidence interval for this situation [18]. Several of these methods approximate the binomial distribution with a normal distribution, which is only reasonable for large n, or use other assumptions. To avoid any of these limitations and to keep the methods especially useful for cases of small n (here the probability of observing zero probability bins is the highest), we calculate $CI_{p,k}$ using the conservative yet exact Clopper–Pearson method [19]. It applies a maximum-likelihood approach to estimate $p_{k}$ given the sample S of size n. The required conditions for the method to apply are:

- there are only two possible outcomes of each trial,
- the probability of success for each trial is constant, and
- all trials are independent.

In our case, this is assured by distinguishing the two outcomes “the trial falls within the bin or not”, keeping the sample constant and random sampling.

In practice, there are two convenient ways to compute the confidence Interval $CI_{p,k}$. One way is to look it up, for example, in the original paper by Clopper and Pearson [19], where they present graphics of confidence intervals for different sample sizes n, different numbers of observations x, and different confidence levels. The second option is to compute the intervals using the Matlab function [~, *CI*] = binofit(*x*, *n*, *alpha*) (similar functions exist for R or python) with 1 − *alpha* defining the confidence level. This function uses a relation between the binomial and the Beta-distribution, for more details see e.g., Reference [20] (Section 7.3.4) and Appendix A.

For each k=1,…,K, the NZ estimate ${\hat{p}}_{k}$ is then calculated as the normalized mean value $m_{k}$ of the confidence interval $CI_{p,k}$ according to Equation (1). Normalization with the sum of all $m_{k}$ for k=1,…,K is required to assure that the total sum of probabilities in $\hat{p}$ equals 1. For this reason, the normalized values of ${\hat{p}}_{k}$ can differ a little from the mean of the confidence intervals.

(1) $${\hat{p}}_{k}:=\frac{m_{k}}{\sum_{k}m_{k}},with\;m_{k}=\frac{p_{k,lower}+p_{k,upper}}{2}$$

Two text files with Matlab code (Version 2017b, MathWorks Inc., Natick, MA, USA) of the NZ method and an example application are available as Supplementary Material.

### 2.2. Properties

There are four properties of the NZ estimate ${\hat{p}}_{k}$ that are important for our application:

1. Maximum Entropy by default: For an increasing number of zero probability bins in q, $\hat{p}$ converges towards a uniform distribution. For any zero probability bin $\beta_{k}$ we get $q_{k}=0$, assign the same confidence interval, and, hence, the same NZ estimate. Consequently, estimating p on a size-zero sample results in a uniform distribution $\hat{p}$ with ${\hat{p}}_{k}=1/K$ for all k=1,…,K, which is a maximum-entropy (or minimum-assumption) estimate. For small samples, the NZ estimate is close to a uniform distribution.
2. Positivity: As probabilities are restricted to the interval [0, 1], and it always holds $p_{k,upper}>p_{k,lower}$, the mean value of the confidence interval $CI_{p,k}$ is strictly positive. This also applies to the normalized mean. This is the main property we were seeking to be guaranteed by ${\hat{p}}_{k}$.
3. Convergence: Since $q_{k}$ is a consistent estimator (Reference [21], Section 5.2), it converges in probability towards $p_{k}$ for growing sample size n. Moreover, the ranges of the confidence intervals $CI_{p,k}$ approach zero with increasing sample size n (Reference [19], Figures 4 and 5) and hence, the estimates ${\hat{p}}_{k}$ converge towards $p_{k}$.
4. As described above, due to the normalization in the method, the NZ estimate does not exactly equal the mean of the confidence interval. However, the interval’s mean tends towards $p_{k}$ with growing n and, hence, the normalizing sum in the denominator tends towards one. Consequently, for growing sample size n, the effect of the normalization is of less and less influence.

### 2.3. Illustration of Properties

**Figure 1:**
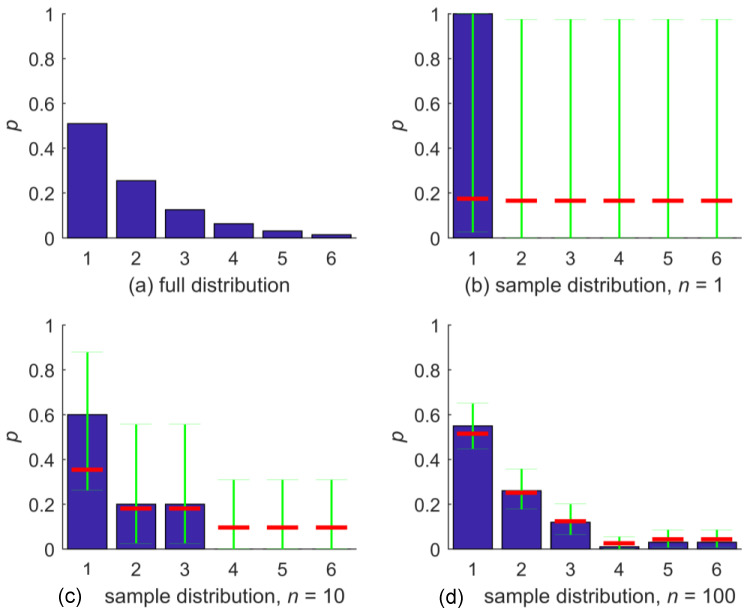
(**a**) Full distribution and (**b**–**d**) samples drawn thereof for different sample sizes *n* shown as blue bars. Green bars are the sample-based confidence intervals on 95% confidence level for bin-occupation probability based on the Clopper–Pearson method, and the red bar is the nonzero estimate for bin-occupation probability.

An illustration of the NZ method and its properties is shown in Figure 1. The first plot, Figure 1a, shows a discrete distribution, constructed for demonstration purposes such that it covers a range of different bin probabilities. Possible outcomes are the six integer values {1, 2,…,6}, where p(1)=0.51 and all further probabilities are half of the previous, such that p(6)=0.015. Figure 1b shows a random sample of size one taken from the distribution; here, the sample took the value “1”. The BC estimator q for the distribution p for outcomes {1,…,6} is shown with blue bars. Obviously, we encounter the problem of zero-probability bins here. In the same plot, the confidence intervals for the bin-occupation probability based on the Clopper–Pearson method on 95% confidence level are shown in green. Due to the small sample size, the confidence intervals are almost the same for all outcomes, and so is the NZ estimate for bin-occupation probability shown in red. Altogether, the NZ estimate is close to a uniform distribution, which is the maximum entropy estimate, except that the bin-occupation probability for the observed outcome “1” is slightly higher than for the others: The NZ estimate of the distribution is $\hat{p}=\left(0.1737,\;0.1653,\;0.1653,\;0.1653,\;0.1653,\;0.1653\right)$. We can also see that the positivity requirement for bin occupation probability is met.

In Figure 1c,d, BC and NZ estimates of the bin-occupation probability are shown for random samples of size 10 and 100, respectively. For sample size 10, the BC method still yields three zero-probability bins, which are filled by the NZ method. The NZ estimates for this sample still gravitate towards a uniform distribution (red bars) but, due to the increased sample size, to a lesser degree than before. For sample size 100, both the BC and the NZ distribution estimate of bin-occupation probability closely agree with the full distribution, which illustrates the convergence behavior of the NZ method. Compared to the size-10 sample, the Clopper–Pearson confidence intervals for the bin-occupation probabilities have narrowed considerably, and, as a result, the NZ estimates are close to those from BC.

## 3. Comparison to Alternative Distribution Estimators

### 3.1. Test Setup

How does the NZ method compare to established distribution estimators that also assure NZ bin-occupation probabilities? We address this question by applying various estimation methods to several types of distributions. In the following, we will explain the experimental setup, the evaluation method, the estimation methods, and the distributions used.

We start by taking samples S of size n by i.i.d. picking (random sampling with replacement) from each distribution p. Each estimation method we want to test applies this sample to construct a NZ distribution estimate $\hat{p}$. The (dis-)agreement of the full distribution with each estimate is measured with the Kullback–Leibler divergence as shown in Equation (2).

(2) $$D_{KL}(p||q)=\underset{\beta \in X}{\sum }p\left(\beta \right)log_{2}\frac{p\left(\beta \right)}{q\left(\beta \right)}$$

with $D_{KL}$: Kullback–Leibler divergence [bit]; p: reference distribution; q: distribution estimate; X: set taking discrete values ${ß}_{k}$ (“bins”) for k=1,…,K.

Note that, for our application, the full distribution of the variable is the reference p, since the observations actually occur according to this distribution; the distribution estimate q is derived from the sample and is our assumption about the variable. We chose Kullback–Leibler divergence as it conveniently measures, in a single number, the overall agreement of two distributions, instead of focusing on particular aspects, e.g., particular moments. Kullback–Leibler divergence is also zero if and only if the two distributions are identical, while, for instance, two distributions with identical mean and variance can still differ in higher moments.

We tested sample sizes from n=1 to 150, increasing n in steps of one. We found an upper limit of 150 to be sufficient for two reasons: Firstly, the problem of zero-probability bins due to the combined effect of sampling variability and limited sample size mainly occurs for small sample sizes; secondly because, for large samples, the distribution estimates by the tested methods quickly become indistinguishable. To eliminate effects of sampling variability, we repeated the sampling for each sample size 1000 times, calculated Kullback–Leibler divergence for each and then took the average. As a result, we get mean Kullback–Leibler divergence as a function of sample size, separately for each estimation method and test distribution.

**Figure 2:**
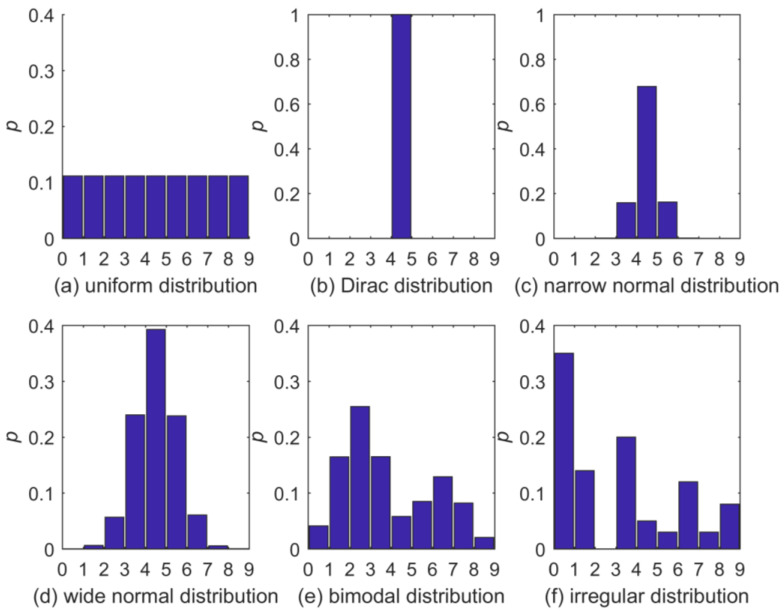
Test distributions: (**a**) Uniform, (**b**) Dirac, (**c**) narrow normal, (**d**) wide normal, (**e**) bimodal and (**f**) irregular. Possible outcomes are divided in nine bins of uniform width. Note that for (**b**,**c**), the y-axis limit is 1.0, but for all others it is 0.4.

The six test distributions are shown in Figure 2. We selected them to cover a wide range of shapes. Please note that two of the distributions, Figure 2b,f, actually contain bins with zero p. It may seem that, in such a case, the application of a distribution estimator assuring NZ p’s is inappropriate; however, in our targeted scenarios (e.g., comparison of two distributions via Kullback–Leibler divergence), it is the zero p’s due to limited sample size that we need to avoid, while we accept the adverse effect of falsely correcting true zeros. If the existence and location of true-zero bins were known a priori, this knowledge could be easily incorporated in the distribution estimators discussed here to only produce actual NZ p’s.

Finally, we selected a range of existing distribution estimators to compare to the NZ method:

1. BC: The full probability distribution is estimated by the normalized BC frequencies of the sample taken from the full data set. This method is just added for completeness, and as it does not guarantee NZ bin probabilities its divergences are often infinite, especially for small sample sizes.
2. Add one (AO): With a sample taken from the full distribution, a histogram is constructed. Any empty bin in the histogram is additionally filled with one counter before converting it to a pdf by normalization. The impact of each added counter is therefore dependent on sample size.
3. BAY: This approach to NZ bin-probability estimation places a Dirichlet prior on the distribution of bin probabilities and updates to a posterior distribution in the light of the given sample via a multinomial-likelihood function [22]. We use a flat uniform prior (with the Dirichlet distribution parameter alpha taking a constant value of one over all bins) as a maximum-entropy approach, which can be interpreted as a prior count of one per bin. Since the Dirichlet distribution is a conjugate prior to the multinomial-likelihood function, the posterior again is a Dirichlet distribution with analytically known updated parameters. We take the posterior mean probabilities as distribution estimate and, for our choice of prior, they correspond to the observed bin counts increased by the prior count of one. Hence, BAY is very similar to AO with the difference that a count of one is added to all bins instead of only to empty bins; like for AO, the impact of the added counters is dependent on sample size. Like the NZ method, BAY is by default a strictly positive and convergent maximum-entropy estimator (see Section 2.2).
4. Add p (AP): With a sample taken from the full distribution, a histogram is constructed and normalized to yield a pdf. Afterwards, each zero-probability bin is filled with a small probability mass (here: 0.0001) and the entire pdf is then renormalized. Unlike in the “AO” procedure, the impact of each probability mass added is therefore virtually independent of n.
5. KDS: We used the Matlab Kernel density function ksdensity as implemented in Matlab R2017b with a normal kernel function, support limited to [0, 9.001], which is the range of the test distributions, and an iterative adjustment of the bandwidth: Starting from an initially very low value of 0.05, the bandwidth (and with it the degree of smoothing across bins) was increased in 0.001 increments until each bin had NZ probability. We adopted this scheme to avoid unnecessarily strong smoothing while at the same time guaranteeing NZ bin probabilities.
6. NZ: We applied the NZ method as described in Section 2.1.

### 3.2. Results and Discussion

**Figure 3:**
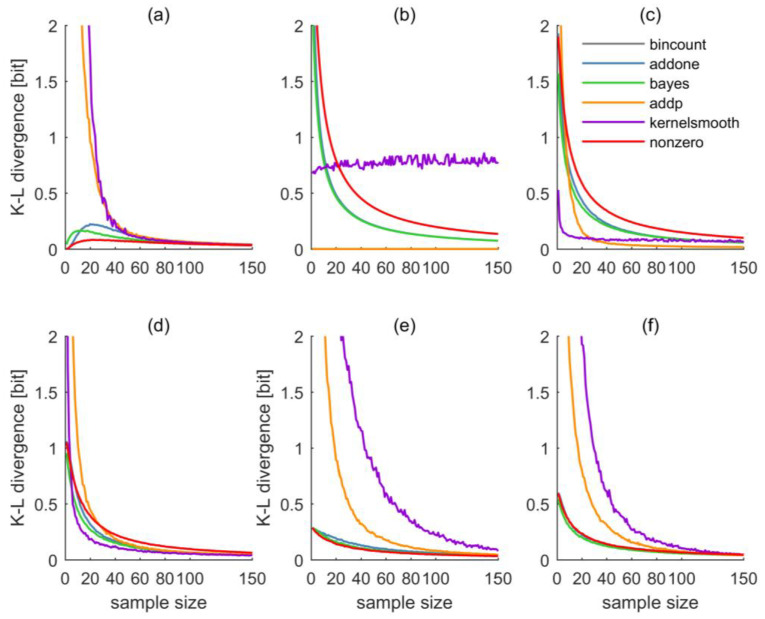
(**a**) Kullback–Leibler divergences of test distributions uniform, (**b**) Dirac, (**c**) narrow normal, (**d**) wide normal, (**e**) bimodal, and (**f**) irregular and size-n samples thereof. Sample-based distribution estimates are based on bin counting (grey), “Add one counter” (blue), “Bayesian” (green), “Add probability” (orange), “Kernel-density smoothing” (violet), and the “nonzero method” (red). In all plots except (**b**), the “bincount” line is invisible as its divergence is infinite, and in plot (**b**) it is invisible as it is zero and almost completely overshadowed by the “addp” line. In plots (**b,f**), the “addone” line is almost completely overshadowed by the “bayes” line. For better visibility, all *y*-axes are limited to a maximum divergence of 2 bit, although this limit is sometimes clearly exceeded for small sample sizes.

The results of all tests, separately for each test distribution and estimation method are shown in Figure 3. We will discuss them first individually for each distribution and later summarize the results.

For the uniform distribution as shown in Figure 2a, the corresponding Kullback–Leibler divergences are shown in Figure 3a. For small sample sizes up to approximately 40, both AP and KDS show very large divergences, AO, BAY, and NZ perform considerably better, with a slight advantage of NZ. This order clearly reflects the methods’ different estimation strategies, and how capable they are to reproduce a uniform distribution: For small sample sizes, both AP and KDS will maintain “spiky” distribution estimates, while AO, BAY, and NZ gravitate towards uniform distribution or maximum-entropy estimates. For larger sample sizes, beyond 80, the performance differences among the methods quickly vanish. For the small sample sizes as shown in the figure, the BC approach was still frequently afflicted with zero-probability bins, resulting in infinite divergence.

Quite expectedly, the relative performance of the estimators for the Dirac distribution (Figure 2b and Figure 3b) is almost opposite from the uniform distribution. BC shows zero and AP almost-zero divergence for all sample sizes. The reason is that even a very small sample from a Dirac distribution yields a perfect estimate of the full distribution, and both methods do not interfere much with this estimate (in fact, BC not at all). AO and BAY show almost identical performance, NZ is similar but slightly worse. All of them show high divergences for small samples and a gradual decrease with sample size. The reason lies in the methods’ tendency towards a uniform spreading of probabilities, which is clearly unfavorable if the true distribution is a Dirac. Interestingly, the KDS estimator performs constantly poorly over the entire range of sample sizes, which can be explained by its tendency of locally distributing probability mass around the BC estimate. In particular, as the kernel function was chosen to be normal, the observed divergence of about 0.8 bit corresponds to the divergence of a Dirac and a normal distribution extending over the nine bins covering the codomain.

For the narrow normal distribution as shown in Figure 2c and Figure 3c, obviously the normal kernel of KDS is of advantage, such that, for small sample sizes, divergence is smaller than for any other estimator. The performance of AP varies greatly with sample size: For small samples it is poor, for sample sizes beyond about thirty it scores best. AO and BY are almost identical, NZ is similar to them but shows worse performance; altogether it is the worst estimator. Beyond sample sizes of about 80, all methods perform almost equally well, except for BC, whose divergence is infinite due to the occasional occurrence of zero-probability bins.

For the wide normal distribution as shown in Figure 2d and Figure 3d, KDS remains the best estimator except for very small sample sizes. AO and BAY are similar and perform better than the NZ method. AP performs worst for sample sizes smaller about thirty; for larger samples, NZ performs worst.

For the bimodal distribution as shown in Figure 2e and Figure 3e things look differently: Both KDS and AP show poor performance even for large sample sizes; AO, BAY, and NZ are almost indistinguishable and they perform well even for small sample sizes.

Finally, results for the application to the irregular distribution as shown in Figure 2f are shown in Figure 3f. As this distribution shows no pattern in the distribution of probabilities across the value domain, any approach assuming a particular shape of pattern (like KDS) will have difficulties, at least for small sample sizes. This is clearly reflected in the large divergences of KDS. Interestingly, AP also struggles to reproduce the irregular distribution, but not because of the absence of a probability pattern across the value domain, but because filling a bin that has zero probability due to chance with always the same small probability mass, irrespective of the sample size, here is less effective than filling it with an adaptive probability mass as done by AO. AO, BAY, and NZ, again, perform almost equally well and better than the other methods (BC again has infinite divergences).

## 4. Summary and Conclusions

We started by describing use cases that involve estimation of discrete distributions with the additional requirement that all bins of the estimated distribution should have NZ probabilities. As the standard BC approach does not guarantee this, we proposed an alternative approach based on the Clopper–Pearson method, which makes use of the binominal distribution. Based on the BC-distribution estimate, confidence intervals for bin-occupation probability are calculated. The mean of each confidence interval is a strictly positive estimator of the true bin-occupation probability and is convergent with increasing sample size. For small samples, it converges towards a uniform distribution, i.e., the method effectively applies a maximum-entropy approach. We compared the capability of this “NZ” method to estimate different distributions (uniform, Dirac, narrow normal, wide normal, bimodal, and irregular) based on i.i.d. samples thereof of different sizes. For comparison, we applied four alternative estimators guaranteeing NZ bin probabilities (adding one counter to each empty bin of the sample histogram, a BAY approach applying a Dirichlet prior, and a multinomial likelihood function, adding a small probability to the sample pdf, and KDS). We measured the agreement of the distributions and their respective estimates via Kullback–Leibler divergence. The most obvious result is that the relative performance of the estimators strongly depends on whether their estimation strategy matches the shape of the test distribution or not. So if the latter is known (or can be reasonably guessed) a priori, a case-specific choice should be made. However, if this is not the case, it is reasonable to select an estimator that performs, on average, well across all distributions. For the range of distributions tested here, this could be either the straightforward method of adding one counter to each empty bin of a sample histogram, the BAY method, or the NZ method. As could be expected by their design, the first two show almost identical behavior and performance. The NZ method is similar to them in overall performance and its dependency of performance on sample size, except that it performs better for close-to-uniform distributions and worse for spiky distributions. Each of the three methods (AO, NZ, and BAY) is straightforward to implement and computationally inexpensive, so from a practical viewpoint, there is no preference for one method or the other. The main differences are in the formal background: The “AO” method lacks a formal justification; the NZ method is based on a statistical/frequentist background, while the BAY method applies a BAY perspective. Although the NZ and the BAY methods are formulated in different formal frameworks, they are in fact very similar (both are maximum-entropy estimators by construction), and so is their performance. Their main differences are that the NZ method applies the binominal distribution to evaluate each bin separately, while the BAY method applies the multinomial distribution simultaneously to all bins. The second difference is that the NZ method uses the normalized mean of the confidence interval of bin probability as the best estimate of bin probability; the BAY method uses the posterior mean. An advantage of the NZ and the BAY over the AO method is that, in addition to the distribution estimate, they also provide confidence intervals that offer additional avenues of analysis or conditioning. An additional advantage of the BAY method is that it offers adaptability: If a priori estimates of the distribution shape are available, they can be considered via the choice of the Dirichlet distribution parameter alpha. Overall, users may make a choice according to the formal setting they are most comfortable with.

## Supplementary Materials

The following are available online at http://www.mdpi.com/1099-4300/20/8/601/s1. Two text files with Matlab code (version 2017b) are available as supplementary material. File “f_NonZero_method.m” is a function to compute the NZ estimate of a pdf given its histogram, file “apply_NonZero_method.m” is an application example calling the function. The files are also available on Github at https://github.com/KIT-HYD/NonZero_method (accessed on 13 August 2018).

## Appendix A. The Clopper–Pearson Method

Let Χ be a discrete random variable that may take values in $\chi =\left\{{ß}_{1},\ldots ,{ß}_{k}\right\}$ and $S_{n}=S_{n}\left(X\right)=\left\{x_{1},\ldots ,x_{n}\right\}$ a set of realisations of X. For each $\beta_{k}\in \chi$, we estimate the probability $p\left(\beta_{k}\right)$ of an observation from X to fall into bin $\beta_{k}$ by the BC probability

(A1) $$q\left({ß}_{k}\right)=\frac{\left|\{x\in S_{n}\left(X\right)|x={ß}_{k}\}\right|}{n}.$$

The aim is to develop a confidence interval $CI=\left[p_{L},p_{U}\right]$ for $p\left({ß}_{k}\right)$ to the confidence level (1–α)=95%.

We derive a confidence interval for each $p_{k}:=p\left({ß}_{k}\right)$ individually; for the sake of readability, we neglect the index k in the following and only write $p:=p_{k}$, $q:=q_{k}$. Let $Y=|\{x\in S_{n}\left(X\right)|x={ß}_{k}\}|$ be the random variable that counts all the observations in $S_{n}$ which actually fall into bin ${ß}_{k}$. This Y has a binominal distribution with parameters n and p, Y~Bin(n,p), i.e., for y∈{0,…,n}

(A2) $$\mathbb{P}\left(Y=y\right)=\left(\begin{array}{c} n\\ y \end{array}\right)p^{y}{\left(1-p\right)}^{n-y}$$

Based on the result Y = y (“The bin ${ß}_{k}$ is observed y times in $S_{n}$”), we want to give a 95% confidence interval for p around q.

**Property** **A1.** *Consider a random variable*Y~Bin(n,p)*. Based on the observation of*y*successes out of the*n*trials, a confidence interval for*p*to the confidence level*(1–α)*is given by*$CI=\left[p_{L},p_{U}\right]$, such that

(A3) $$\overset{n}{\underset{j=y}{\sum }}\left(\begin{array}{c} n\\ j \end{array}\right)p_{L}^{j}(1-p_{L}{)}^{n-j}=\frac{\propto }{2}$$

(A4) $$\overset{y}{\underset{j=0}{\sum }}\left(\begin{array}{c} n\\ j \end{array}\right)p_{U}^{j}(1-p_{U}{)}^{n-j}=\frac{\propto }{2}$$

**Proof.** These confidence intervals were introduced by Reference [19]. The following proof is based on Reference [20] (Theorem 7.3.4). For any p∈(0,1) and corresponding Y~Bin(n,p), take the largest $h_{1}\left(p\right)\in \mathbb{N}$ with $\mathbb{P}\left[Y\leq h_{1}\left(p\right)\right]\leq \alpha /2$ and the smallest $h_{2}\left(p\right)\in \mathbb{N}$ with $\mathbb{P}\left[Y\geq h_{2}\left(p\right)\right]\leq \alpha /2$. For these values we get

(A5) $$\mathbb{P}\left[h_{1}\left(p\right)<Y<h_{2}\left(p\right)\right]=1-\mathbb{P}\left[Y\leq h_{1}\left(p\right)orY\geq h_{2}\left(p\right)\right]=1-\propto$$

Since the functions $h_{1},h_{2}:\left(0,1\right)\to \mathbb{N}$ are monotonically increasing discontinuous step functions, we can define $g_{1}\left(y\right):=\min\left\{h_{1}^{-1}\left(\left\{y\right\}\right)\right\}$ as the minimal p, such that $h_{1}\left(p\right)=y$; analogously, $g_{2}\left(y\right):=\max\left\{h_{2}^{-1}\left(\left\{y\right\}\right)\right\}$ is the maximal p with $h_{2}\left(p\right)=y$. With these definitions, the events $\left[h_{1}\left(p\right)<Y<h_{2}\left(p\right)\right]$ and $\left[g_{2}\left(Y\right)<p<g_{1}\left(Y\right)\right]$ are equivalent, so we have

(A6) $$\mathbb{P}\left[g_{2}\left(Y\right)<p<g_{1}\left(Y\right)\right]\geq 1-\propto$$

We have shown that for the observation Y=y and for $p_{L}:=g_{2}\left(y\right)$, $p_{U}:=g_{1}\left(y\right)$, $CI=\left[p_{L},p_{U}\right]$ is a (1–α) confidence interval for p. For Y~Bin(n,p) it holds

(A7) $$\mathbb{P}\left[Y\leq h_{1}\left(p\right)\right]\leq \frac{\propto }{2}⟺\overset{h_{1}\left(p\right)}{\underset{j=0}{\sum }}\left(\begin{array}{c} n\\ j \end{array}\right)p^{j}(1-p{)}^{n-j}\leq \frac{\propto }{2}$$

Hence, the upper limit $p_{U}$ fulfills the following equation:

(A8) $$\overset{h_{1}\left(p_{u}\right)}{\underset{j=0}{\sum }}\left(\begin{array}{c} n\\ j \end{array}\right)p_{U}^{j}(1-p_{U}{)}^{n-j}\leq \frac{\propto }{2}$$

With $h_{1}\left(p_{U}\right)=h_{1}\left(g_{1}\left(y\right)\right)=y$ and since we are looking for the smallest-possible upper bound, $p_{U}$ has to satisfy (A4). Similarly, we can show that $p_{L}$ has to satisfy (A3). Of course, for y=0, we have $p_{L}=0$ and easily compute

(A9) $$\overset{0}{\underset{j=0}{\sum }}\left(\begin{array}{c} n\\ j \end{array}\right)p_{U}^{j}{\left(1-p_{U}\right)}^{n-j}=\frac{\propto }{2}⟺{\left(1-p_{U}\right)}^{n}=\frac{\propto }{2}⟺p_{U}=1-\sqrt[{n}]{\frac{\propto }{2}}$$

In the same way, for y=n, we get $CI=\left[\sqrt[{n}]{\frac{\propto }{2}},1\right]$. For all other y=1,…,n−1, there is no such easy closed solution to compute. Reference [19] and others give tables that list the solutions for these equations for some n∈ℕ. For our purposes (i.e., for use in Matlab codes), however, it is more convenient to use the relation between the binomial and the beta distribution to compute the confidence interval limits $p_{L}$ and $p_{U}$ [23,24]. ☐

**Property** **A2** (see e.g., Reference [25], Equation (3.37), Chapter 3). *For a random variable* Y~Bin(n,p) *and* y∈{1,…,n−1}, *it holds*

(A10) $$\mathbb{P}\left(Y\geq y\right)=I_{p}\left(y,n-y+1\right),$$

(A11) $$\mathbb{P}\left(Y\leq y\right)=1-I_{p}\left(y+1,n-y\right),$$

*where* $I_{x}\left(a,b\right)=\frac{1}{B\left(a.b\right)}\int_{0}^{x}t^{a-1}{\left(1-t\right)}^{b-1}dt$ *is the regularized incomplete beta function with the beta function* $B\left(a,b\right)=\int_{0}^{1}t^{a-1}{\left(1-t\right)}^{b-1}dt$.

**Proof.** First of all, with the property

(A12) $$I_{1-x}\left(a,b\right)=1-I_{x}\left(b,a\right)$$

for $a,b\in {\mathbb{R}}_{>0}$, x∈(0,1) (e.g., (olver), Equation (8.17.4)), it holds

(A13) $$\mathbb{P}\left(Y\leq y\right)=1-I_{p}\left(y+1,n-y\right)=I_{1-p}\left(n-y,y+1\right).$$

Moreover, for the beta function with a,b∈ℕ, we have

(A14) $$B\left(a,b\right)=\frac{\left(a-1\right)!\left(b-1\right)!}{\left(a+b-1\right)!}$$

(e.g., Reference [26], Equation (5.12.1)). Using this, Equation (A13) can be derived by integration by parts:

(A15) $$\begin{array}{ll} I_{1-p}\left(n-y,y+1\right) & =\frac{n!}{\left(n-y-1\right)!y!}\int_{0}^{1-p}t^{n-y-1}{\left(1-t\right)}^{y}dt\\ & =\frac{n!}{\left(n-y-1\right)!y!}\left({\left[\frac{1}{n-y}t^{n-y}{\left(1-t\right)}^{y}\right]}_{0}^{1-p}+\int_{0}^{1-p}\frac{1}{n-y}t^{n-y}y{\left(1-t\right)}^{y-1}dt\right)\\ & =\frac{n!}{\left(n-y\right)!y!}p^{y}{\left(1-p\right)}^{n-y}+\frac{n!}{\left(n-y\right)!\left(y-1\right)!}\int_{0}^{1-p}t^{n-y}{\left(1-t\right)}^{y-1}dt\\ & =\left(\begin{array}{c} n\\ y \end{array}\right)p^{y}{\left(1-p\right)}^{n-y}+I_{1-p}\left(n-\left(y-1\right),\left(y-1\right)+1\right). \end{array}$$

For y=0, the claim simplifies to

(A16) $$I_{1-p}\left(n,1\right)=\frac{n!}{\left(n-1\right)!}\int_{0}^{1-p}t^{n-1}dt=n{\left[\frac{1}{n}t^{n}\right]}_{0}^{1-p}={\left(1-p\right)}^{n}=\mathbb{P}\left(Y\leq 0\right)$$

so Equation (A11) follows by induction. From there, Equation (A10) follows directly

(A17) $$\begin{array}{ll} \mathbb{P}\left(Y\geq y\right)=1-\mathbb{P}\left(Y<y\right)= & 1-\mathbb{P}\left(Y\leq y-1\right)=1-\left[1-I_{p}\left(\left(y-1\right)+1,n-\left(y-1\right)\right)\right]\\ & =I_{p}\left(y,n-y+1\right). \end{array}$$

The beta distribution Beta(a,b), $a,b\in {\mathbb{R}}_{>0}$, is defined by its probability density function (for x∈(0,1))

(A18) $$f\left(x;a,b\right)=\frac{1}{B\left(a,b\right)}x^{a-1}{\left(1-x\right)}^{b-1}$$

and on the interval (0,1), the cumulative distribution function is

(A19) $$F\left(x;a,b\right)=I_{x}\left(a,b\right).$$

Hence, from all the considerations above results the following theorem, which we use to compute the Clopper–Pearson confidence intervals for the binominal distribution parameter p. ☐

**Theorem** **A1.** *Consider the random variable*X*with values in*χ={ß1,…,ßk}*. If bin*${ß}_{k}$*is observed*$y=|\{x\in S_{n}\left(X\right)|x={ß}_{k}\}|$*times in a data set*$S_{n}\left(X\right)$*of length*n*, the*(1−α)*confidence interval for the probability of bin*${ß}_{k}$*is*$CI=\left[p_{L},p_{U}\right]$, *where*

(a) *for*y=1,…,n−1:
  (i) $p_{L}$*is the*α/2*quantile of the beta distribution*Beta(y,n−y+1),
  (ii) $p_{U}$*is the*1−α/2*quantile of the beta distribution*Beta(y+1,n−y);
(b) *for*y=0:
  (i) $p_{L}=0$,
  (ii) $p_{U}=1-\sqrt[{n}]{\frac{\propto }{2}}$;
(c) *for*y=n:
  (i) $p_{L}=\sqrt[{n}]{\frac{\propto }{2}}$,
  (ii) $p_{U}=1$.
